# Peritoneal Incretin Deficiency and Tirzepatide as a Multi-Axis Adjuvant Hypothesis in Treatment-Refractory Endometriosis: A Mechanistic Framework Linking Metabolism, Immunity, Fibrosis, and Nociception

**DOI:** 10.3390/ijms27135678

**Published:** 2026-06-24

**Authors:** Leonardo Jacobsen, Diogo Pinto da Costa Viana, Graciela Morgado Folador, Eduardo Schor, Adriana Luckow Invitti

**Affiliations:** 1 Brazilian Society for Research and Teaching in Medicine (SOBRAPEM), São Paulo 01318-901, Brazil; pinto.viana@unifesp.br; 2 Department of Gynecology, Escola Paulista de Medicina, Federal University of São Paulo (EPM-UNIFESP), São Paulo 04024-002, Brazil; schor@unifesp.br (E.S.); adriana.invitti@unifesp.br (A.L.I.); 3 Brazilian Society of Personalized Medicine (SBMP), São Paulo 04552-050, Brazil; dra.gracielamorgado@gmail.com

**Keywords:** endometriosis, tirzepatide, GLP-1 receptor agonists, peritoneal incretin deficiency, AMPK, NLRP3 inflammasome, TGF-β1, metabolic reprogramming, chronic pelvic pain, immunometabolism

## Abstract

Endometriosis is increasingly recognized as a chronic systemic disorder extending beyond the classical estrogen-dependent paradigm, integrating metabolic, immune, fibrotic, and nociceptive pathways that sustain lesion persistence and refractory pelvic pain. We propose a mechanistic, translational hypothesis in which tirzepatide, a dual glucose-dependent insulinotropic polypeptide (GIP) and glucagon-like peptide-1 (GLP-1) receptor agonist, may modulate four interconnected pathological axes of refractory endometriosis—Warburg-type metabolic reprogramming with lactate accumulation, peritoneal immune dysfunction, NF-κB/NLRP3/TGF-β1-driven inflammatory–fibrotic remodeling, and persistent nociceptive sensitization—through three convergent molecular nodes: AMPK-associated signaling, GLP-1 receptor activity in peritoneal macrophages and spinal microglia, and the NF-κB/NLRP3/TGF-β1 axis. Particular emphasis is placed on the concept of “peritoneal incretin deficiency”, characterized by reduced peritoneal GLP-1 concentrations and increased expression of incretin-degrading proteases. This concept currently rests on a single, non-replicated case–control study, and the broader mechanistic chain is supported largely by indirect evidence extrapolated from adjacent inflammatory, metabolic, and neuroimmune disease models rather than by endometriosis-specific data. Direct experimental or clinical validation in endometriosis-specific models is currently absent. Accordingly, this article represents a hypothesis-generating framework rather than evidence of established efficacy, or a clinical treatment recommendation, intended to guide future mechanistic and prospective clinical investigation of incretin-based modulation as a potential adjunctive strategy in refractory endometriosis.

## 1. Introduction

Endometriosis is a chronic systemic disease that affects approximately 10% of women of reproductive age, and its pathophysiology extends beyond the classical estrogen-dependent paradigm. Metabolic, immunological, inflammatory, and nociceptive alterations interact as an integrated network of multi-axis dysregulation that sustains lesion persistence, fibrotic remodeling, and chronic pelvic pain [1,2,3]. Despite this complexity, current therapeutic strategies remain predominantly centered on sequential hormonal suppression—including combined oral contraceptives, continuous progestins, and gonadotropin-releasing hormone (GnRH) analogues—and on surgical excision, in accordance with current ESHRE management guidelines [4]. Although these approaches provide substantial benefit for many patients, refractory disease and postoperative symptom persistence remain frequent clinical challenges, suggesting that suppression of ovarian hormonal signaling alone may incompletely address the broader systemic immunometabolic and neuroinflammatory architecture of endometriosis.

A historical barrier to recognizing the systemic dimension of endometriosis has been the body mass index (BMI) paradox. Epidemiological studies have consistently associated the disease with lower BMI, a finding that historically reinforced the perception that endometriosis predominantly affects lean women. More recent evidence suggests that this phenotype may partially reflect reverse-causality mechanisms, in which chronic low-grade inflammation, altered hepatic and adipose tissue metabolism, and neuroendocrine adaptations associated with active disease contribute to weight loss and relative leanness [1,5]. Despite a frequently normal or reduced BMI, women with endometriosis may exhibit isolated hyperinsulinemia, atherogenic lipid profiles, oxidative stress, and increased prevalence of metabolic syndrome independent of clinically recognized obesity [6,7,8]. Recent stratified analyses using the #ENZIAN classification have additionally shown that patients with normal or low BMI report significantly higher intensity of chronic pelvic pain compared to those with overweight or obesity, reinforcing the clinical relevance of disease manifestations in lean phenotypes [9]. Together, these observations support the interpretation that immunometabolic dysregulation represents an intrinsic component of the disease rather than a simple consequence of excess adiposity. They additionally raise the possibility that therapeutic modulation of these pathways may provide benefit beyond body weight reduction alone.

Against this dysmetabolic background, a recent biochemical observation has introduced a molecular clue of particular translational relevance. In a case–control study including 54 patients with endometriosis and 30 controls, glucagon-like peptide-1 (GLP-1) concentrations were significantly reduced in the peritoneal fluid of affected patients (*p* = 0.009), in parallel with increased expression of the GLP-1-degrading protease neprilysin (CD10) in peritoneal macrophages [10]. This finding, although derived from a single non-replicated cohort, suggests the existence of a localized state of impaired incretin signaling—conceptualized here as “peritoneal incretin deficiency”—within an anatomical compartment central to the pathophysiology of the disease. The observation raises the mechanistic possibility that pharmacological restoration of GLP-1 receptor (GLP-1R) agonism by molecules resistant to local proteolytic degradation could partially recover an endogenously compromised regulatory pathway.

Tirzepatide, a 39-amino-acid synthetic polypeptide with dual agonism at glucose-dependent insulinotropic polypeptide (GIP) and GLP-1 receptors, was originally developed for the treatment of type 2 diabetes mellitus and obesity but displays pleiotropic effects that extend beyond glycemic and weight control. Recent systematic reviews and meta-analyses have demonstrated reductions in systemic inflammatory markers, favorable modulation of lipid and endothelial profiles, and protective effects across cardiovascular, renal, hepatic, and neurological systems [11,12,13]. In parallel, preliminary observational data from patients with endometriosis exposed to GLP-1 receptor agonists (GLP-1RAs) have reported self-reported improvement in menstrual and non-menstrual pelvic pain, with symptom recurrence after treatment discontinuation [14]. Although these findings remain exploratory and cannot establish causality, they provide an early clinical signal consistent with the broader immunometabolic and neuroactive profile attributed to incretin-based therapies. The present framework also extends the immunometabolic rationale previously developed by our group in the distinct biological context of lipedema [15] to endometriosis, in which peritoneal incretin deficiency adds a disease-specific compartmental dimension absent from the lipedema model.

The convergence among the emerging concept of peritoneal incretin deficiency, the multi-axis architecture of endometriosis pathophysiology, and the pleiotropic immunometabolic profile of tirzepatide provides a biologically plausible rationale for systematic investigation of this agent in refractory disease. The present mechanistic hypothesis proposes that tirzepatide may simultaneously modulate four interconnected pathological axes of endometriosis—metabolic reprogramming, peritoneal immune dysfunction, chronic inflammatory–fibrotic remodeling, and nociceptive sensitization—through convergence on three shared molecular nodes. Importantly, the hypothesis is positioned as complementary rather than substitutive to current standard therapies and is intended to support future experimental and prospective clinical investigation rather than immediate therapeutic implementation.

## 2. Background

### 2.1. Endometriosis as a Systemic Immunometabolic Disease

The conceptual reframing of endometriosis as a systemic rather than strictly pelvic disorder has emerged from convergent clinical, epidemiological, immunometabolic, and neurobiological evidence [1,2]. Ectopic and stromal endometrial cells exhibit features consistent with Warburg-type metabolic reprogramming, including preferential aerobic glycolysis under normoxic conditions, increased HIF-1α and pyruvate dehydrogenase kinase 1 (PDK1) activity, and consequent peritoneal lactate accumulation [16,17,18]. Single-cell transcriptomic analyses further identified HIF-1- and AMPK-associated pathways among the most differentially regulated metabolic networks in ectopic lesions, particularly within stromal, endothelial, and perivascular cellular populations [16,17,18]. In parallel, the peritoneal immune compartment displays impaired macrophage phagocytic competence, reduced natural killer cell cytotoxicity, and sustained mast cell activation [19,20,21,22,23].

Importantly, inflammatory and metabolic alterations associated with endometriosis do not appear confined to the pelvic cavity. Population studies have reported independent associations between endometriosis and metabolic syndrome, hypercholesterolemia, systemic arterial hypertension, type 2 diabetes mellitus, and coronary artery disease, including in patients without clinically recognized obesity [5,7,8,24]. Genome-wide association data further support the systemic nature of endometriosis, identifying shared genetic architecture with multiple pain and inflammatory comorbidities and reinforcing the interpretation of the disease as part of a broader spectrum of chronic inflammatory disorders [25]. Although causality remains incompletely established, these findings support the interpretation that systemic metabolic and inflammatory dysregulation may represent intrinsic components of disease biology rather than secondary consequences of excess adiposity alone. Concurrently, increased expression of transforming growth factor beta 1 (TGF-β1), alpha smooth muscle actin (α-SMA), and type I collagen contributes to adhesional remodeling and deep infiltrating disease, establishing overlap with profibrotic pathways observed in other chronic inflammatory conditions [26,27].

At the nociceptive level, chronic pelvic pain in endometriosis involves both peripheral and central mechanisms, including visceral hypersensitivity, peritoneal neuroinflammation, spinal sensitization, microglial activation, and overlap with disorders of central pain amplification such as irritable bowel syndrome [28,29,30]. Together, these observations support the interpretation that endometriosis operates as a structurally multi-axis disorder involving interconnected metabolic, immune, fibrotic, and neuroimmune dimensions. Under this framework, therapeutic strategies focused predominantly on ovarian hormonal suppression may incompletely address the broader biological substrate contributing to refractory disease.

### 2.2. Tirzepatide: Pharmacological Architecture and Pleiotropic Profile

Tirzepatide is a 39-amino-acid synthetic polypeptide with dual agonism at GIP and GLP-1 receptors, currently approved for the treatment of type 2 diabetes mellitus and obesity [12,13]. Structural modifications confer resistance to degradation by DPP-4, resulting in prolonged half-life and stable once-weekly pharmacokinetic exposure. Simultaneous activation of GIP and GLP-1 receptors appears to modulate intracellular pathways involving cyclic adenosine monophosphate (cAMP), protein kinase A (PKA), phosphatidylinositol 3-kinase (PI3K)/Akt signaling, and AMPK-associated networks. Downstream, these converge on mammalian target of rapamycin (mTOR), HIF-1α, NF-κB, the NLRP3 inflammasome, and TGF-β1 signaling [12,13,24].

Beyond glycemic control and weight reduction, incretin-based therapies have demonstrated broader anti-inflammatory and organ-protective effects across experimental and clinical settings. Meta-analyses involving tirzepatide reported reductions in TNF-α, IL-6, IL-1β, and high-sensitivity C-reactive protein (hsCRP), together with favorable modulation of lipid profile, endothelial function, and systemic arterial pressure [11]. Preclinical evidence additionally suggests that incretin-based therapies may exert biologically relevant effects within the central nervous system, including modulation of microglial activation, attenuation of oxidative stress, and activation of neurotrophic pathways involving brain-derived neurotrophic factor (BDNF) and tropomyosin receptor kinase B (TrkB) [12,13]. However, the extent to which these findings translate directly to human neuroinflammatory conditions remains uncertain.

Collectively, these observations describe a pharmacological profile whose molecular targets overlap with several pathways implicated in endometriosis pathophysiology. AMPK has emerged as a potentially important regulator of metabolic and inflammatory signaling; GLP-1 receptors have been identified in peritoneal macrophages [10] and spinal microglia [31]; and modulation of the NF-κB/NLRP3/TGF-β1 axis may theoretically influence inflammatory, fibrotic, and nociceptive processes simultaneously. Although the available evidence derives from heterogeneous experimental systems and partially overlapping disease models, this convergence provides the mechanistic basis for the hypothesis developed in the following sections. Specifically, we propose that dual GIP/GLP-1 receptor agonism by tirzepatide may modulate the four interconnected pathological axes of refractory endometriosis through three partially shared molecular nodes: AMPK-associated signaling, GLP-1 receptor activity in peritoneal immune cells and central nervous system microglia, and the NF-κB/NLRP3/TGF-β1 axis. The following sections develop each axis in turn, with the explicit objective of generating testable mechanistic predictions rather than implying established therapeutic efficacy.

## 3. Mechanism 1: Modulation of Warburg-Type Metabolic Reprogramming

The persistence of endometriotic lesions within a hypoxic and inflammatory peritoneal microenvironment appears closely linked to adaptive metabolic remodeling. Ectopic endometrial cells exhibit features consistent with Warburg-type metabolic reprogramming, including preferential aerobic glycolysis and enhanced conversion of pyruvate into lactate under conditions not exclusively explained by oxygen deprivation [18]. Such metabolic adaptation may confer adaptive advantages by limiting excessive mitochondrial reactive oxygen species (ROS) generation and reducing susceptibility to oxidative stress-associated apoptosis [18,32].

Single-cell transcriptomic analyses further identified differential regulation of HIF-1α- and AMPK-associated pathways within stromal, endothelial, and perivascular cellular populations of ectopic lesions compared with paired eutopic endometrium [16,27]. Mechanistically, this network appears to involve TGF-β1-associated stabilization of HIF-1α, activation of PDK1, restriction of pyruvate entry into the tricarboxylic acid cycle, and increased lactate dehydrogenase A (LDHA) activity, culminating in sustained lactate accumulation [17,18]. Importantly, these alterations have been described across both lean and overweight phenotypes, supporting the interpretation that altered cellular energetics may represent an intrinsic component of disease biology rather than a simple consequence of obesity-related metabolic dysfunction [6,8].

Taken together, these observations identify metabolic reprogramming as a potentially relevant therapeutic dimension in endometriosis, although the extent to which these pathways operate independently of systemic metabolic status and adiposity remains incompletely established.

Peritoneal lactate accumulation may represent more than a passive metabolic byproduct and instead function as a biologically active intermediary linking inflammatory, immune, fibrotic, and nociceptive pathways. Experimental evidence suggests that elevated lactate concentrations may influence macrophage polarization through epigenetic mechanisms including histone lysine lactylation, thereby contributing to dysfunctional immune responses within the peritoneal environment [17,22]. In parallel, lactate appears capable of reinforcing autocrine TGF-β1 signaling and sustaining glycolytic and fibrotic feedback loops [27,33]. Additional evidence suggests that lactate may also influence neuronal excitability through monocarboxylate transporters expressed in peripheral and central nociceptive pathways [28,29].

Within this context, AMPK has emerged as an integrative regulatory node linking metabolic, inflammatory, and mitochondrial signaling. AMPK activation has been associated with inhibition of mTOR, destabilization of HIF-1α signaling, reduced expression of PDK1 and LDHA, partial restoration of oxidative metabolism, and attenuation of lactate accumulation [17,18]. Experimental proof-of-concept studies support the therapeutic relevance of this axis: dichloroacetate reduced lesion size and peritoneal lactate levels in murine models [18,34]; metformin attenuated inflammatory signaling in preclinical studies [23]; and sitagliptin reduced TNF-α, IL-6, cyclooxygenase 2, prostaglandin E2, and HIF-1α expression in human endometrial stromal cells [35]. Together, these findings support the interpretation that metabolic modulation may represent a pharmacologically actionable dimension of endometriosis biology.

Within the present hypothesis, tirzepatide may influence metabolic reprogramming through upstream modulation of AMPK-associated signaling via dual GIP/GLP-1 receptor agonism. Experimental studies in cardiometabolic and neurological models suggest that simultaneous activation of GIPR and GLP-1R may amplify cAMP/PKA-associated AMPK signaling compared with selective GLP-1 receptor agonism alone [12,13]. Additional indirect support derives from studies involving liraglutide in endometrial cancer cells, in which AMPK activation was associated with increased autophagic and apoptotic signaling [36]; importantly, endometrial cancer biology differs fundamentally from endometriotic lesion biology, and direct extrapolation should therefore be considered speculative.

The prolonged half-life and resistance of tirzepatide to DPP-4 degradation may hold particular relevance in endometriosis given the dysregulation of incretin-degrading proteases reported in peritoneal macrophages from affected patients [10]. Under this framework, modulation of AMPK-associated signaling could theoretically reproduce the metabolic effects outlined above and, in addition, contribute to indirect suppression of the TGF-β1/HIF-1α/lactate feedback loop through interactions with NF-κB- and NLRP3-associated inflammatory pathways. Such effects could potentially alter metabolic conditions favoring ectopic lesion persistence and inflammatory amplification.

Nevertheless, important limitations remain. The single most consequential translational gap concerns receptor characterization: functional expression of GLP-1R and GIPR in eutopic and ectopic endometriotic tissue has not yet been systematically demonstrated at the protein level, as opposed to transcript (mRNA) detection alone. Moreover, no direct experimental study has shown tirzepatide-induced metabolic modulation in human endometriotic lesions. Demonstrating functional receptor protein in the target tissue is therefore a prerequisite for the entire framework rather than a peripheral caveat. Consequently, the present framework should be interpreted as a mechanistic and translational hypothesis derived from convergent evidence across partially overlapping biological systems rather than as evidence of established therapeutic efficacy.

## 4. Mechanism 2: Modulation of Peritoneal Immune Dysfunction

From an immunological perspective, endometriosis is increasingly understood as a condition in which the peritoneal microenvironment exhibits impaired capacity to eliminate refluxed endometrial tissue and may instead support lesion implantation, vascularization, and persistence [19,20,37]. Peritoneal macrophages from affected patients display altered expression of inhibitory receptors such as signal regulatory protein alpha (SIRP-α) and CD200R, together with reduced expression of scavenger receptors including CD36, findings consistent with dysfunctional phagocytic activity toward ectopic endometrial cells [20,22]. In parallel, macrophage polarization appears shifted toward reparative and profibrotic phenotypes associated with neoangiogenesis and tissue remodeling [37]. Recent single-cell transcriptomic analyses of the endometriotic niche have further identified distinct macrophage phenotypes associated with prodisease and proresolving functions, providing a more granular framework that extends beyond the classical M1/M2 dichotomy [38]. Additional immune alterations include reduced natural killer cell cytotoxicity, functional exhaustion of peritoneal T lymphocytes, expansion of regulatory T-cell populations, and sustained mast cell activation within the peritoneal compartment [21,23,39].

Within this context, the observation by Krasnyi et al. that peritoneal GLP-1 concentrations are reduced in patients with endometriosis (*p* = 0.009), in association with increased expression of the incretin-degrading protease neprilysin (CD10) (*p* = 0.044) in peritoneal macrophages, represents a finding of particular mechanistic interest [10]. In the same study, expression of dipeptidyl peptidase 4 (DPP-4/CD26) was not significantly increased between groups but correlated positively with the reduced concentrations of GLP-1, glucagon, and visfatin. The upregulation of a GLP-1-degrading protease within this inflammatory compartment raises the possibility of localized impairment of incretin signaling, conceptualized here as “peritoneal incretin deficiency”. It must be emphasized, however, that this concept is currently supported by a single non-replicated case–control study (54 patients, 30 controls), in which multiple peptide hormones (ghrelin, glucagon, visfatin, GLP-1) were simultaneously reduced; alternative explanations including increased local consumption, transcriptional downregulation, or extracellular vesicle sequestration cannot be excluded. Independent replication of peritoneal GLP-1 measurements in larger cohorts is therefore the rate-limiting validation step for this hypothesis.

Experimental studies have begun to delineate molecular pathways through which GLP-1 receptor signaling may influence macrophage behavior. In murine peritoneal macrophages and RAW264 cells, stimulation of GLP-1R with exendin-4 reduced lipopolysaccharide-induced macrophage migration and attenuated secretion of TNF-α, IL-6, and IL-1β [40]. Mechanistically, these effects were associated with reduced phosphorylation of IκBα, inhibition of NF-κB nuclear translocation, and consequent suppression of pro-inflammatory cytokine transcription. Importantly, receptor silencing experiments substantially attenuated these effects, supporting a receptor-dependent mechanism involving canonical cAMP/PKA-associated signaling pathways. These data establish functional, protein-level GLP-1R signaling in murine macrophages; corresponding evidence in human endometriotic tissue, at either transcript or protein level, remains to be established.

Additional studies suggest that GLP-1R activation may also influence macrophage polarization and mitochondrial homeostasis through interactions involving signal transducer and activator of transcription 3 (STAT3) and AMPK signaling [10,22,23]. Importantly, the inflammatory mediators most consistently modulated by GLP-1 receptor signaling, notably IL-6 and IL-1β, are recurrently reported as elevated in the peritoneal fluid of women with endometriosis. These same cytokines are secreted by endometrial stromal cells at concentrations significantly higher than in control subjects, even under hormonal modulation [8,30]. This convergence between incretin-modulated cytokines and the inflammatory signature of the endometriotic peritoneal microenvironment provides a disease-specific biological rationale for considering GLP-1 receptor-mediated effects within endometriosis pathophysiology rather than as a generic anti-inflammatory mechanism. Under this framework, incretin signaling may therefore exert dual effects within the peritoneal compartment: attenuation of inflammatory cytokine production and modulation of macrophage functional phenotype. However, this interpretation requires explicit caution. The classical anti-inflammatory reparative M2 phenotype induced experimentally by GLP-1R signaling may not be equivalent to—and could conceivably overlap with—the dysfunctional profibrotic M2-like phenotype observed in endometriosis. Whether incretin-mediated modulation would restore effective immune clearance without simultaneously amplifying profibrotic signaling is unknown and represents a critical experimental question. Indeed, this concern is empirically grounded: in murine models of endometriosis, macrophage depletion attenuates lesion fibrosis, whereas adoptive transfer of M2a macrophages restores and exacerbates fibrotic progression, and IL-4-driven M2a polarization promotes TGF-β1 production and epithelial–mesenchymal transition [20,22]. Future studies should characterize the specific macrophage subtype (M2a/M2b/M2c) induced by tirzepatide in human endometriotic peritoneal fluid before any translational implication is drawn.

Within the present hypothesis, tirzepatide may offer theoretical advantages in the context of impaired peritoneal incretin signaling. Resistance to DPP-4 degradation and prolonged pharmacokinetic exposure may be particularly relevant in a compartment characterized by dysregulated expression of incretin-degrading proteases [10,13]. In contrast to DPP-4 inhibitors, which depend on endogenous GLP-1 availability, dual GIP/GLP-1 receptor agonism provides direct receptor stimulation and may therefore partially circumvent the limitations imposed by local proteolytic degradation and reduced endogenous incretin concentrations.

The proposed mechanistic consequences of this signaling include attenuation of NF-κB-associated inflammatory activity in peritoneal macrophages, reduction in local TNF-α, IL-6, and IL-1β production, modulation of macrophage metabolic homeostasis through AMPK-associated pathways, and indirect effects on natural killer cell and mast cell activation secondary to altered cytokine signaling. The hypothesis further raises the possibility that incretin-based modulation may contribute to partial restoration of functional immune surveillance within the peritoneal environment. Nevertheless, these predictions remain speculative and have not yet been directly validated in human endometriotic tissue or in experimental models specifically evaluating tirzepatide exposure.

## 5. Mechanism 3: Modulation of Inflammatory and Fibrotic Pathways

The systemic dimension of endometriosis supports the interpretation that the disease may operate, at least partially, as a chronic inflammatory and fibrotic disorder extending beyond the pelvic compartment. Multiple patient cohorts have demonstrated circulating inflammatory signatures characterized by increased serum concentrations of TNF-α, IL-1β, IL-6, homocysteine, and related inflammatory mediators. Importantly, these alterations do not appear entirely attributable to obesity alone. Observational evidence showing normalization of lipid profile and fasting glycemia following surgical treatment of endometriosis, despite minimal changes in body weight or lifestyle, supports the possibility that active disease itself contributes to systemic metabolic dysregulation [5].

Adipokine signaling provides an additional interface between systemic inflammation and lesion biology. Elevated leptin concentrations within serum and peritoneal fluid, together with reduced adiponectin signaling, have been associated with endometriosis [41]. Leptin-mediated activation of the JAK2/STAT3 pathway has been implicated in lesion proliferation, angiogenesis, and persistence, whereas adiponectin appears to exert counter-regulatory anti-inflammatory effects. Analogous patterns of adipose tissue dysfunction, intracrine estrogen excess, and estrogen receptor imbalance have been previously characterized in other hormone-sensitive adipose disorders, supporting the broader concept that adipose tissue may operate as an active endocrine compartment in gynecological disease [42]. Concurrently, increased expression of TGF-β1, α-SMA, and type I collagen contributes to adhesional remodeling and deep infiltrating disease, establishing overlap with profibrotic pathways implicated in pulmonary, hepatic, renal, and cardiac fibrosis [26,27].

An additional dimension of the fibrotic architecture of endometriosis involves epithelial–mesenchymal transition (EMT) and mesenchymal-to-epithelial transition (MET)-like processes, increasingly recognized as central pathways in lesion implantation, persistence, and fibrotic remodeling [43,44]. Experimental evidence in primate models supports the role of EMT together with fibroblast-to-myofibroblast transdifferentiation in disease progression [45]. Notably, EMT has additionally been mechanistically linked to downregulation of progesterone receptor expression in endometriotic lesions, providing a potential molecular bridge between EMT-associated processes and the progesterone resistance phenotype frequently observed in refractory disease [46]. Within this framework, modulation of TGF-β1 and NF-κB signaling, both pathways responsive to incretin-based therapies, may theoretically influence EMT-associated fibrotic remodeling regardless of whether the originating cells derive from retrograde menstruation or coelomic metaplasia.

Additional evidence suggests that chronic psychological stress may further amplify inflammatory and metabolic dysregulation through hypothalamic–pituitary–adrenal axis remodeling and altered sympathetic modulation of peritoneal immune responses [47]. Collectively, these observations support the interpretation that inflammatory, metabolic, neuroendocrine, and fibrotic pathways interact bidirectionally in the progression of refractory disease.

Experimental and translational studies involving incretin-based therapies have identified modulation of NF-κB, the NLRP3 inflammasome, p38 MAPK, JAK/STAT3 signaling, and AMPK-associated pathways across cardiovascular, hepatic, renal, pulmonary, gastrointestinal, cutaneous, and neuroinflammatory models [24]. Several of these pathways are also implicated in endometriosis pathophysiology.

Within the peritoneal environment, activation of the NLRP3 inflammasome in macrophages and mast cells has been associated with release of IL-1β and IL-18, cytokines implicated in persistent inflammatory signaling and nociceptive sensitization [23,29,30]. In parallel, TGF-β1 occupies a central position within the fibrotic architecture of the disease through interactions involving immune polarization, HIF-1α-associated metabolic signaling, and extracellular matrix remodeling.

One of the few direct demonstrations of anti-fibrotic effects of GLP-1 receptor agonism in uterine tissue derives from a murine model of intrauterine synechiae, in which exenatide reduced expression of TGF-β1, α-SMA, and type I collagen while increasing matrix metalloproteinase 9 (MMP-9) expression [48]. Although intrauterine fibrosis and endometriotic fibrosis are biologically distinct entities, partial overlap between their TGF-β1-associated pathways provides translational plausibility for considering incretin-based modulation within fibrotic dimensions of endometriosis. However, direct evidence of human endometriotic fibrosis remains absent.

Clinical studies involving tirzepatide have demonstrated reductions in systemic inflammatory markers, including hsCRP, TNF-α, IL-6, and IL-1β, across populations with obesity and type 2 diabetes mellitus [11]. Meta-analytic data indicating approximately 32% reductions in hsCRP concentrations suggest that incretin-based therapies may exert measurable systemic anti-inflammatory effects in humans. Preclinical studies additionally reported attenuation of inflammatory signaling through modulation of TLR4/NF-κB/NLRP3 and PI3K/Akt-associated pathways in models of cardiomyopathy, nephropathy, and neuroinflammation [12,13].

Because several of these pathways are also implicated in endometriosis-associated inflammation and fibrosis, tirzepatide may theoretically influence interconnected inflammatory and fibrotic processes within the disease. Potential mechanisms include attenuation of NF-κB-associated cytokine signaling, modulation of inflammasome activity, indirect suppression of TGF-β1-associated fibrotic remodeling, and partial interference with leptin-associated proliferative signaling. Of particular relevance to endometriosis, the TGF-β1/SMAD and NF-κB axes potentially modulated by incretin signaling are the same pathways that drive EMT and fibroblast-to-myofibroblast transdifferentiation in endometriotic lesions [43,44,45,46]; their attenuation could therefore plausibly interfere with lesion stabilization and progression regardless of the originating paradigm, whether retrograde implantation or coelomic metaplasia. Under this framework, incretin-based modulation could potentially affect both pelvic inflammatory activity and broader systemic metabolic-inflammatory manifestations observed in subsets of refractory patients.

Importantly, these proposed mechanisms remain inferential. Most available evidence derives from heterogeneous experimental systems or diseases with partially overlapping inflammatory biology rather than from direct investigation in endometriosis itself. Consequently, the extent to which anti-inflammatory and anti-fibrotic effects observed in cardiometabolic or non-gynecological models translate to human endometriosis remains uncertain and requires dedicated mechanistic and prospective clinical investigation.

## 6. Mechanism 4: Modulation of Nociceptive Sensitization

Chronic pelvic pain in endometriosis is unlikely to be fully explained by local peritoneal inflammation alone. Increasing evidence supports a multilevel nociceptive architecture involving both peripheral and central mechanisms [49,50,51,52]. Peripheral contributors include visceral hypersensitivity, peritoneal neuroinflammation, and intralesional neuroangiogenesis driven by nerve growth factor and inflammatory cytokines; peripheral neurogenic changes, including increased nerve fiber density, altered distribution of sensory and autonomic fibers, and upregulation of neurotrophic factors, have been extensively characterized in endometriotic lesions and their vicinity [49]. Beyond the spinal dorsal horn, neuroimaging and experimental studies implicate supraspinal structures in endometriosis-associated central sensitization, including the thalamus, insula, amygdala, and hippocampus, where altered functional connectivity and regional microglial and astroglial activation have been reported [53]. Importantly, the experimental evidence for GLP-1 receptor engagement in pain modulation is anatomically restricted to the spinal dorsal horn, and no data currently address incretin signaling within these supraspinal circuits [28,29,30]. Bidirectional crosstalk between nociceptive neurons and immune cells represents a critical mechanism in the development and maintenance of chronic pain conditions and provides a unifying framework for understanding the inflammatory amplification of pain in endometriosis [54]. In parallel, endometriosis frequently overlaps with disorders characterized by central pain amplification, including irritable bowel syndrome and fibromyalgia-like hyperalgesic phenotypes [50,51]. More recent conceptual developments increasingly recognize endometriosis-associated pain within the broader framework of chronic pain biology, integrating nociceptive, neuropathic, and nociplastic mechanisms across heterogeneous patient phenotypes [51,52].

Metabolomic analyses of serum and endometrial tissue have additionally demonstrated increased glutamate concentrations together with alterations in glycine, serine, and threonine metabolism, findings consistent with enhanced excitatory signaling potentially relevant to nociceptive sensitization [55]. Importantly, in a substantial subset of patients, pain persists after surgical lesion removal. This suggests that chronic nociceptive remodeling may, in some cases, persist independently of the original peripheral lesion burden, and may therefore require therapeutic strategies targeting central neuroimmune pathways in addition to local pelvic pathology.

Over the past two decades, GLP-1 receptor signaling has emerged as a potential modulatory pathway in experimental pain biology. In preclinical models, intrathecal administration of exendin-4 reduced pain hypersensitivity across inflammatory, neuropathic, diabetic neuropathic, and bone cancer pain paradigms, with concomitant upregulation of GLP-1R expression in microglial populations within the spinal dorsal horn under inflammatory conditions [31]. Mechanistic studies suggest that GLP-1R activation in spinal microglia may induce release of endogenous beta-endorphin, leading to downstream activation of μ-opioid receptors on spinal interneurons. Importantly, these experimental effects occurred without evidence of altered physiological sensory perception or development of analgesic tolerance within the timeframes evaluated [31].

Subsequent investigations further identified an autocrine signaling loop involving interleukin 10 (IL-10), in which GLP-1R activation stimulates expression of IL-10 and proopiomelanocortin (POMC) through cAMP/PKA/p38β/CREB-associated pathways. IL-10 signaling then appears to amplify beta-endorphin release through STAT3-dependent mechanisms in activated microglia [56]. Together, these findings suggest the existence of a neuroimmune modulatory axis linking incretin signaling, microglial activation, endogenous opioid release, and inflammatory pain modulation.

The broader reproducibility of this framework has been supported by integrative and systematic reviews encompassing experimental and clinical studies involving neuropathic pain, inflammatory pain, osteoarthritis, visceral pain, fibromyalgia, headache disorders, and bone cancer pain [57,58]. However, it is important to emphasize that the majority of mechanistic evidence derives from preclinical models, frequently involving intrathecal administration paradigms rather than systemic exposure, and that direct extrapolation to human pelvic pain conditions remains uncertain.

Human evidence remains indirect but suggestive. Randomized clinical trials have demonstrated reductions in pain-related outcomes in idiopathic intracranial hypertension treated with exenatide [59] and in knee osteoarthritis treated with semaglutide [60]. Additional observational studies have reported improvement in headache burden in idiopathic intracranial hypertension under GLP-1RA exposure [61]. In a large real-world cohort involving patients with knee osteoarthritis and type 2 diabetes mellitus, exposure to GLP-1RAs was associated with reduced incidence of joint surgery, an observation interpreted as potentially reflecting disease-modifying and anti-inflammatory effects extending beyond weight reduction alone [62]. Collectively, these findings support the possibility of clinical translation of GLP-1-associated neuroimmune modulation across conditions sharing inflammatory and sensitization-related pain mechanisms.

Within the present framework, tirzepatide may theoretically influence nociceptive sensitization in endometriosis at both peripheral and central levels. An important caveat must precede this discussion: most preclinical evidence supporting GLP-1R-mediated central analgesia derives from intrathecal administration of exendin-4, which achieves spinal drug exposure several orders of magnitude higher than systemic subcutaneous administration of tirzepatide in humans. Whether systemic tirzepatide achieves cerebrospinal fluid and dorsal horn concentrations sufficient to engage microglial GLP-1R in a clinically meaningful magnitude remains uncharacterized and represents a critical translational gap. Peripherally, attenuation of inflammatory signaling through modulation of NF-κB-associated cytokine pathways—including TNF-α, IL-6, IL-1β, nerve growth factor, and TGF-β1—could potentially reduce neurogenic inflammation and sensitization of perilesional nociceptive fibers [10,40,48]. In parallel, modulation of metabolic reprogramming and reduction in lactate accumulation may theoretically influence neuronal excitability mediated by monocarboxylate transporters, thereby connecting metabolic and nociceptive dimensions of the disease [17,29].

At the central level, GLP-1R activation in spinal microglia may theoretically modulate nociceptive processing through partially overlapping mechanisms involving endogenous beta-endorphin release and attenuation of intracellular inflammatory signaling pathways [31,56,57]. Preclinical evidence from neurodegenerative disease models further suggests that incretin-based therapies may achieve biologically relevant central nervous system exposure [12,13], although the extent of central penetration and relevance to human endometriosis remains incompletely characterized. This component rests entirely on intrathecal rodent paradigms and currently lacks any endometriosis-specific or systemic-exposure data. The central opioidergic axis (microglial GLP-1R signaling leading to β-endorphin release and downstream μ-opioid receptor activation) is therefore designated here as the most exploratory element of the present framework, of lower evidentiary status than the peripheral anti-inflammatory and metabolic mechanisms.

Preliminary observational signals from patients with endometriosis exposed to GLP-1RAs are compatible with this framework. In an international observational survey, menstrual and non-menstrual pelvic pain, low back pain, and lower-limb pain were among the symptoms most frequently reported as improved during exposure to GLP-1RAs, with symptom recurrence after treatment discontinuation [14]. However, these findings remain exploratory, self-reported, and non-controlled, and therefore cannot establish causality.

Taken together, the available evidence supports the hypothesis that incretin-based modulation may influence neuroimmune and nociceptive pathways relevant to chronic pelvic pain in endometriosis. Nevertheless, direct evidence specifically involving systemic tirzepatide exposure in experimental or clinical endometriosis models is currently absent. Consequently, the proposed analgesic framework should be interpreted as a biologically plausible but still unvalidated translational hypothesis requiring dedicated mechanistic and prospective clinical investigation.

## 7. Synthesis and Clinical Positioning

### 7.1. Convergent Mechanistic Architecture: Three Shared Molecular Nodes

The mechanisms discussed in the previous sections should not be interpreted as isolated biological compartments but rather as interconnected components of a broader immunometabolic and neuroinflammatory network. Integrated analysis of these pathways suggests three partially convergent molecular nodes through which incretin-based therapies may theoretically influence multiple dimensions of endometriosis pathophysiology.

The first involves AMPK-associated signaling, which has emerged as a regulator of metabolic reprogramming, mitochondrial homeostasis, inflammatory signaling, macrophage function, and microglial responses [17,24,56,57]. The second involves GLP-1 receptor signaling identified in peritoneal macrophages [10,40], spinal dorsal horn microglia [31,58], and dorsal root ganglion neurons [56,58], raising the possibility of coordinated modulation across peripheral immune and central nociceptive compartments. The third involves the NF-κB/NLRP3/TGF-β1 signaling network, implicated across inflammatory, fibrotic, metabolic, and nociceptive pathways relevant to endometriosis and modulated by incretin-based therapies in multiple cardiometabolic and neuroinflammatory models [5,12,13,47].

Importantly, this interpretation does not imply uniform responsiveness across disease phenotypes. Endometriosis is biologically heterogeneous, encompassing distinct inflammatory, fibrotic, hormonal, and nociceptive profiles. Nevertheless, partial convergence of these pathways raises the possibility that systemic incretin-based modulation may influence multiple pathogenic processes through a limited number of shared molecular interfaces.

Current therapeutic strategies predominantly target ovarian hormonal signaling, whereas the present hypothesis proposes that persistent symptoms in refractory disease may additionally involve broader immunometabolic, inflammatory, fibrotic, and neuroimmune mechanisms. Under this perspective, tirzepatide is not proposed as a replacement for standard therapies but rather as a potential adjunctive investigational strategy directed toward biological processes insufficiently addressed by hormonal suppression alone. Table 1 summarizes the principal mechanistic axes discussed throughout this article, their associated pathogenic alterations, the proposed convergent molecular nodes, and the hypothesized biological effects potentially associated with incretin-based modulation.

### 7.2. Implications for Clinical Investigation: Adjuvant Positioning and the Weight-Loss Tension

The present hypothesis is explicitly positioned outside the concept of therapeutic substitution. Hormonal therapies and surgical approaches remain established components of endometriosis management and provide meaningful benefit for many patients. Tirzepatide is proposed only as a potential adjunctive investigational strategy for refractory phenotypes characterized by persistent symptoms despite conventional treatment.

Such phenotypes frequently involve manifestations extending beyond classical estrogen-dependent models, including chronic fatigue, visceral hypersensitivity, overlap with irritable bowel syndrome, widespread pain amplification, and subclinical inflammatory or metabolic dysfunction [1,24,56,58]. These features partially overlap with pathways theoretically influenced by incretin-based therapies and therefore support a rationale for translational investigation.

An important methodological tension concerns the relationship between immunometabolic modulation and body weight reduction. Although several pathological dimensions discussed throughout this article may operate partially independently of obesity, tirzepatide remains strongly associated with clinically significant weight loss in cardiometabolic populations. Consequently, potential translational application in normal-weight patients raises concerns regarding nutritional status, lean body mass preservation, reproductive physiology, and bone homeostasis.

Preliminary observational findings suggest that symptomatic improvement during exposure to GLP-1RAs may occur across heterogeneous BMI categories, including normal-weight individuals [14]. However, these observations remain exploratory and do not establish whether potential clinical effects derive primarily from body weight reduction, systemic metabolic modulation, anti-inflammatory signaling, neuroimmune effects, or combinations thereof.

Future translational studies should incorporate prospective body composition monitoring (DEXA, bioimpedance), nutritional surveillance, predefined criteria for unintended weight loss, and analytical strategies capable of partially dissociating immunometabolic effects from adiposity reduction. Patients with concurrent overweight or obesity (BMI ≥ 27 kg/m^2^) may represent the most methodologically favorable population for initial randomized investigation, while the relevance of incretin-based modulation in lean refractory phenotypes remains an open translational question. Candidate weight-independent biomarkers of response include peritoneal lactate, hsCRP-to-weight ratios, macrophage polarization profiles, and serum/peritoneal inflammatory cytokine signatures.

**Figure 1:**
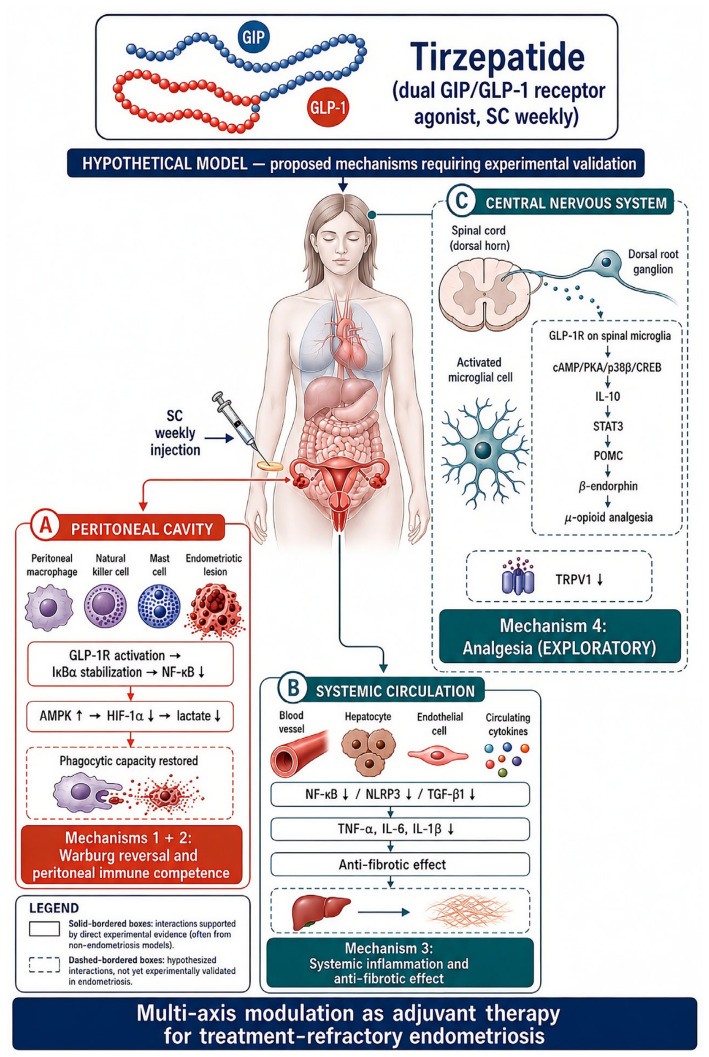
Proposed multi-axis framework of incretin-based modulation in treatment-refractory endometriosis. Tirzepatide, a dual GIP/GLP-1 receptor agonist, is hypothesized to influence interconnected immunometabolic, inflammatory, fibrotic, and nociceptive pathways across three biological compartments relevant to endometriosis pathophysiology. In the peritoneal compartment, GLP-1R- and AMPK-associated signaling may modulate NF-κB-dependent inflammatory pathways, glycolytic metabolic remodeling involving HIF-1α and lactate accumulation, and immune signaling involving macrophages, natural killer cells, and mast cells (Mechanisms 1 and 2). In the systemic compartment, modulation of the NF-κB/NLRP3/TGF-β1 signaling network may theoretically influence inflammatory and fibrotic pathways associated with cytokine production, α-SMA expression, collagen deposition, and metabolic-inflammatory signaling (Mechanism 3). In the central nervous system, GLP-1R signaling in spinal microglia may influence neuroimmune nociceptive processing through IL-10-, STAT3-, and beta-endorphin-associated pathways implicated in central sensitization and inflammatory pain modulation (Mechanism 4). This figure is a hypothetical model: all depicted interactions are proposed mechanisms requiring experimental validation. Solid-bordered boxes denote steps supported by direct experimental evidence, frequently derived from non-endometriosis models; dashed-bordered boxes—together with the entire central nervous system panel (Mechanism 4)—denote hypothesized interactions not yet validated in endometriosis and should be regarded as the most exploratory. Arrows indicate the proposed direction of mechanistic influence between nodes and do not, by themselves, distinguish activation from inhibition; upward (↑) and downward (↓) symbols denote increased or decreased activity or levels of the adjacent molecule. The figure represents a conceptual mechanistic synthesis derived from convergent experimental and translational evidence and should therefore be interpreted as a hypothesis-generating framework rather than evidence of established therapeutic efficacy in endometriosis. This figure was conceptualized and created by the authors using AI-assisted graphical design software (GPT-4o image generation), followed by manual editing and refinement. No copyrighted images or third-party graphical elements were used.

Figure 1 summarizes the proposed multi-axis model integrating peritoneal immune dysfunction, systemic inflammatory–fibrotic signaling, and central neuroimmune nociceptive pathways potentially influenced by incretin-based modulation.

### 7.3. Phenotype-Specific Plausibility

The plausibility of the proposed framework is unlikely to be uniform across endometriosis subtypes. Superficial peritoneal endometriosis, ovarian endometrioma, and deep infiltrating endometriosis (DIE) differ substantially in their immune, metabolic, hormonal, fibrotic, and neurogenic signatures, and these differences are expected to translate into heterogeneous responsiveness to incretin-based modulation [1,63,64]. Single-cell transcriptomic profiling of the three subtypes within the same patients has confirmed that macrophage, stromal, and fibroblastic signatures diverge markedly by lesion type [65].

Superficial peritoneal disease represents the most plausible candidate. It is dominated by active inflammation and disordered peritoneal macrophage function, and it is precisely within the peritoneal compartment that reduced GLP-1 and increased CD10 protease expression were originally documented [10]. Because peritoneal lesions carry a comparatively immature fibrotic burden, the anti-inflammatory and metabolic dimensions of the present framework have here the greatest modifiable substrate.

Ovarian endometriomas occupy an intermediate position. Their biology is driven predominantly by local estrogen metabolism and iron-related oxidative stress, so that any incretin-mediated benefit would likely be indirect, for example through reduction in peripheral aromatization and metabolic-inflammatory signaling, rather than through direct lesion modification. Direct GLP-1, GIP, or receptor data in this phenotype are absent.

Deep infiltrating endometriosis represents the least favorable phenotype for a lesion-modifying effect. It is characterized by mature fibrosis with myofibroblastic transdifferentiation and dense hyperinnervation, features associated with established structural remodeling and central pain sensitization [49,66]. Pharmacological reversal of established fibrosis is difficult across organ systems, and antifibrotic strategies act more consistently on active fibrogenesis than on consolidated extracellular matrix [66]. A peripherally acting metabolic agent would therefore be expected to attenuate ongoing inflammatory and fibrogenic activity rather than to regress an established fibromuscular nodule, and would not be expected to address central sensitization.

Taken together, the biological plausibility of incretin-based modulation appears graded across phenotypes, being highest in inflammatory, metabolically active, fibrotically immature peritoneal disease and lowest in fibrosis-dominant DIE. This phenotype-aware framing reinforces rather than weakens the hypothesis, since it aligns the proposed molecular target with the compartment in which incretin dysregulation was actually observed. It also defines a clear experimental priority: characterization of GLP-1R, GIPR, and incretin-degrading protease expression across all three subtypes, since no phenotype-stratified incretin data currently exist.

## 8. Limitations and Future Directions

The present hypothesis has important limitations that must be explicitly acknowledged. Most importantly, there is currently no direct experimental or clinical evaluation of tirzepatide—or any GLP-1RA—in endometriosis models specifically assessing pelvic pain, lesion regression, peritoneal fibrosis, or immune alterations within the peritoneal microenvironment. The mechanistic framework proposed here therefore derives from triangulation across partially overlapping biological systems, including murine models of intrauterine fibrosis [48], inflammatory and neuropathic pain paradigms [31,56,58], and human translational observations in conditions such as idiopathic intracranial hypertension and osteoarthritis [59,60]. Most foundational studies examining GLP-1R signaling in peritoneal macrophages [40] and spinal microglia [31] were additionally conducted in murine systems, and the extent to which these findings translate to human endometriotic tissue remains unknown.

Two further caveats deserve emphasis. First, as discussed in detail in Section 7.3, endometriosis is a biologically heterogeneous disorder encompassing superficial peritoneal, ovarian, and deep infiltrating phenotypes with distinct inflammatory and fibrotic signatures [1], and uniform responsiveness across these subtypes should not be assumed. Second, three factors together delimit the scope of any clinical translational hypothesis: the single-cohort nature of the evidence underlying the “peritoneal incretin deficiency” concept [10]; the incomplete pharmacokinetic characterization of tirzepatide within the peritoneal cavity and central nervous system [12,13]; and the clinical tension between immunometabolic modulation and unintended weight loss in normal-weight patients (Section 7.2). Replication of the peritoneal incretin deficiency finding in independent cohorts and characterization of GLP-1R/GIPR expression in eutopic and ectopic endometrial tissue should therefore be considered the rate-limiting validation step before patient-level investigation.

### 8.1. Reproductive and Sex-Specific Safety Considerations

Because the target population predominantly includes women of reproductive age, several sex-specific safety dimensions deserve dedicated attention before translational investigation is undertaken. First, human pregnancy safety data on tirzepatide remain limited, and animal studies have raised concerns about potential reproductive toxicity; a wash-out interval of at least two months prior to attempted conception is currently recommended by regulatory bodies. Second, women with endometriosis frequently present with normal or low BMI and chronic pain-related deconditioning; tirzepatide-induced weight loss could plausibly precipitate or aggravate sarcopenia and amenorrhea in this subgroup. Inflammatory myosteatosis and dynapenia have been documented in adjacent chronic inflammatory conditions affecting women, supporting the biological plausibility of accelerated muscle dysfunction under conditions of systemic inflammation combined with weight loss [67]. Third, prevalence of disordered eating and body image distress is increased in chronic pelvic pain populations, and incretin-based therapies may exacerbate these conditions. Fourth, gastroparesis associated with GLP-1 receptor agonism may reduce systemic absorption of oral hormonal therapies in general. While most regulatory attention has focused on the contraceptive efficacy of combined oral contraceptives, recent pharmacokinetic evidence indicates that tirzepatide co-administration substantially reduces Cmax (approximately 59–66%) and AUC (approximately 20–21%) of orally administered hormones [68]. This pharmacokinetic interaction is potentially relevant not only for contraceptive efficacy but also for therapeutic progestogen exposure in patients receiving continuous progestins, dienogest, or micronized progesterone for endometriosis-related indications. The clinical implication is that observed changes in symptomatic control during tirzepatide exposure may, in some cases, reflect altered pharmacokinetics of concomitant hormonal therapy rather than intrinsic effects on the disease itself. Future prospective investigation should therefore systematically document concomitant hormonal therapy and consider stratified analyses by oral versus non-oral routes of progestogen delivery. Finally, surveillance for known class-specific adverse effects—including pancreatitis, severe gastrointestinal symptoms, and thyroid C-cell signals identified in preclinical models—remains mandatory. Future prospective investigation therefore requires rigorous informed consent procedures, systematic contraceptive counseling, baseline body composition assessment, and predefined criteria for treatment discontinuation in the event of unintended weight loss exceeding clinically defined thresholds.

A further consideration specific to this population concerns fertility itself, since many women with endometriosis actively desire conception. Incretin receptors are not inert within the reproductive axis: GIP and GLP-1 receptors are expressed in the pituitary, ovary, and uterus, and their genetic deletion alters estrous cyclicity and reduces fertility in animal models [69]. Pharmacological modulation of these receptors could therefore exert reproductive effects that remain uncharacterized in endometriosis. The direction of such effects appears context-dependent: in obesity and polycystic ovary syndrome, GLP-1 receptor agonists improve ovulatory function and reproductive outcomes, largely through weight reduction and direct hypothalamic stimulation of luteinizing hormone secretion [70]. However, because women with endometriosis frequently present with normal or low body weight, this weight-mediated reproductive benefit may not apply, whereas the potential to perturb gonadal signaling persists. In the absence of human pregnancy safety data and given the recommended pre-conception washout interval, incretin-based therapy should be regarded as incompatible with active attempts at conception, and any prospective investigation in this population must incorporate explicit fertility counseling and contraceptive safeguards.

### 8.2. Testable Predictions and Research Priorities

To distinguish this framework from a speculative narrative, its central components are stated as falsifiable predictions that define a sequential experimental agenda. The most proximal and decisive test is whether functional GLP-1R and GIPR protein is detectable by immunohistochemistry in eutopic and ectopic endometrial tissue, with quantifiable differences in receptor density across superficial peritoneal, ovarian, and deep infiltrating phenotypes; absence of receptor protein in ectopic lesions would refute the direct-target premise. The framework further predicts that the reduction in peritoneal GLP-1 and the increased macrophage CD10 expression will be reproduced in independent, phenotype-stratified cohorts, failing which the peritoneal incretin deficiency concept would not be sustained. At the cellular level, it predicts that in ex vivo peritoneal macrophages or endometriotic stromal cells from affected patients, tirzepatide will reduce secretion of IL-6, IL-1β, and TNF-α and lower NF-κB pathway activation in a receptor-dependent manner, that it will attenuate glycolytic markers (LDHA, HIF-1α) and lactate output with measurable AMPK activation, and that dual GIP/GLP-1 receptor agonism will exert greater anti-inflammatory and metabolic effects than selective GLP-1 receptor agonism in the same model. A further prediction, consistent with Section 7.3, is that any anti-fibrotic effect will be confined to active fibrogenesis, with little or no regression of established fibrosis. Collectively, these studies could clarify whether the proposed mechanisms operate directly within endometriotic tissue or indirectly through broader systemic inflammatory and metabolic pathways.

Subsequent translational investigation may include randomized, placebo-controlled clinical studies in refractory endometriosis populations stratified by BMI category and inflammatory phenotype, incorporating prospective assessment of pelvic pain, quality of life, inflammatory biomarkers, body composition (DEXA-based), and metabolic parameters. Within such studies, the framework predicts that any symptomatic benefit will be greatest in inflammation-predominant, superficial or early disease and least in fibrosis-dominant deep infiltrating disease, and that it will be at least partially independent of body-weight reduction. Importantly, future methodological strategies should attempt to partially disentangle immunometabolic and neuroimmune effects from body weight reduction itself, potentially through body-composition–adjusted analyses, predefined nutritional monitoring protocols, and stratification according to baseline adiposity and metabolic phenotype. Candidate biomarkers for future translational investigation include circulating inflammatory mediators, serum and peritoneal lactate concentrations, macrophage polarization profiles, and emerging proteomic signatures associated with incretin-based therapies [71].

Finally, the investigational nature of this framework raises important ethical and regulatory considerations. Tirzepatide does not currently possess regulatory approval for endometriosis treatment, and any use outside formal research protocols should be considered off-label. Investigational use should occur exclusively within institutional review board–approved protocols with explicit risk–benefit communication.

## 9. Conclusions

Endometriosis is increasingly recognized as a complex systemic disorder involving interconnected metabolic, inflammatory, immune, fibrotic, and nociceptive pathways extending beyond classical estrogen-dependent models of disease. Within this context, the present article proposes a mechanistic framework in which tirzepatide, a dual GIP/GLP-1 receptor agonist, may theoretically influence multiple pathological dimensions of refractory endometriosis. These effects would act through partially convergent molecular pathways involving AMPK-associated signaling, GLP-1 receptor-mediated immune and neuroimmune modulation, and the NF-κB/NLRP3/TGF-β1 inflammatory–fibrotic network.

The biological plausibility of this framework derives from the convergence of several independent observations: evidence of altered incretin signaling within the peritoneal environment, the pleiotropic anti-inflammatory and metabolic effects of incretin-based therapies across cardiometabolic and neuroinflammatory systems, and preliminary exploratory signals suggesting symptom modulation in patients exposed to GLP-1RAs. Importantly, however, the present hypothesis remains fundamentally translational and inferential. Direct experimental validation in endometriosis-specific models and prospective clinical investigation are currently absent.

Accordingly, tirzepatide should not be interpreted as a replacement for established hormonal or surgical therapies but rather as a potential adjunctive investigational strategy targeting biological mechanisms that may remain insufficiently addressed in refractory disease. No clinical recommendation can be derived from the present analysis, and no treatment decision should be based on it. The hypothesis must first be validated in dedicated, endometriosis-specific preclinical models before any clinical or translational application can be considered. The translational relevance of this framework will ultimately depend on rigorous mechanistic validation, careful clinical evaluation, and explicit consideration of important unresolved questions, including disease heterogeneity, central nervous system penetration, immune specificity, and the relationship between immunometabolic modulation and body weight reduction.

The most immediate research priority is the immunohistochemical characterization of GLP-1R and GIPR expression in eutopic and ectopic endometriotic tissue across disease phenotypes, together with replication of peritoneal GLP-1 measurements in independent cohorts. These steps represent the rate-limiting validation prerequisites for any subsequent translational investigation.

Taken together, the present framework provides a biologically plausible, mechanistically integrated, and experimentally testable hypothesis supporting future investigation of incretin-based modulation in endometriosis.

## Figures and Tables

**Table 1:** Convergent mechanistic architecture of the proposed hypothesis: four pathological axes of refractory endometriosis, three shared molecular nodes, and hypothesized biological effects of dual GIP/GLP-1 receptor agonism by tirzepatide.

Mechanistic Axis	Principal Pathogenic Alteration	Proposed Convergent Molecular Node	Hypothesized Biological Effect	Key References
Metabolic reprogramming	HIF-1α activation, PDK1 upregulation, lactate accumulation	AMPK	Modulation of glycolytic metabolism and lactate-associated signaling	[16,17,18]
Peritoneal immune dysfunction	Reduced peritoneal GLP-1 signaling, impaired macrophage phagocytosis, inflammatory immune polarization	GLP-1R + AMPK	Modulation of macrophage inflammatory signaling and immune functional competence	[10,20,40]
Systemic inflammation and fibrotic remodeling	Increased inflammatory cytokines, TGF-β1 signaling, α-SMA and collagen expression	NF-κB/NLRP3/TGF-β1	Modulation of inflammatory and fibrotic signaling pathways	[11,24,26,48]
Nociceptive sensitization	Central sensitization, microglial activation, visceral hypersensitivity	Microglial GLP-1R/IL-10/β-endorphin axis	Modulation of neuroimmune nociceptive signaling	[31,56,57,58]

## Data Availability

No new data were created or analyzed in this study. All data discussed in this article are available in the cited primary literature.

## Abbreviations

Akt	Protein kinase B
AMPK	Adenosine monophosphate-activated protein kinase
BDNF	Brain-derived neurotrophic factor
BMI	Body mass index
cAMP	Cyclic adenosine monophosphate
CD10	Cluster of differentiation 10 (neprilysin)
CD36	Cluster of differentiation 36
CD200R	CD200 receptor
CREB	cAMP response element-binding protein
DEXA	Dual-energy X-ray absorptiometry
DPP-4	Dipeptidyl peptidase 4
GIP	Glucose-dependent insulinotropic polypeptide
GIPR	GIP receptor
GLP-1	Glucagon-like peptide-1
GLP-1R	GLP-1 receptor
GnRH	Gonadotropin-releasing hormone
HIF-1α	Hypoxia-inducible factor 1 alpha
hsCRP	High-sensitivity C-reactive protein
IκBα	Nuclear factor kappa B inhibitor alpha
IL-1β/IL-6/IL-10/IL-18	Interleukin 1 beta/6/10/18
JAK2	Janus kinase 2
LDHA	Lactate dehydrogenase A
M1/M2	Macrophage polarization phenotypes
MAPK	Mitogen-activated protein kinase
MMP-9	Matrix metalloproteinase 9
mTOR	Mammalian target of rapamycin
NF-κB	Nuclear factor kappa B
NLRP3	NOD-like receptor family pyrin domain-containing 3 (inflammasome)
p38β	p38 mitogen-activated protein kinase beta
PDK1	Pyruvate dehydrogenase kinase 1
PI3K	Phosphatidylinositol 3-kinase
PKA	Protein kinase A
POMC	Proopiomelanocortin
ROS	Reactive oxygen species
SIRP-α	Signal regulatory protein alpha
STAT3	Signal transducer and activator of transcription 3
TGF-β1	Transforming growth factor beta 1
TLR4	Toll-like receptor 4
TNF-α	Tumor necrosis factor alpha
TrkB	Tropomyosin receptor kinase B
α-SMA	Alpha smooth muscle actin

## References

[1] Taylor H.S., Kotlyar A.M., Flores V.A. (2021). Endometriosis Is a Chronic Systemic Disease: Clinical Challenges and Novel Innovations. Lancet.

[2] Petraglia F., Vannuccini S., Dolmans M.-M., Speciale A.R., Bourdon M., Marcellin L., Donnez J., Chapron C. (2025). The Endocrine Aspects of Endometriosis: An Overview. Eur. J. Endocrinol..

[3] Zondervan K.T., Becker C.M., Missmer S.A. (2020). Endometriosis. N. Engl. J. Med..

[4] Becker C.M., Bokor A., Heikinheimo O., Horne A., Jansen F., Kiesel L., King K., Kvaskoff M., Nap A., Petersen K. (2022). ESHRE Guideline: Endometriosis. Hum. Reprod. Open.

[5] Lavor C.B.H., Viana Júnior A.B., Medeiros F.C. (2024). Analysis of the Metabolic Profile and Comorbidities in Women with Endometriosis Before and After Surgical Treatment. J. Health Biol. Sci..

[6] Chen J.-P., Zhang Y.-Y., Jin J.-N., Ying Y., Song Z.-M., Xu Q.-Q., Tu M.-X., Ye X.-H., Tang H.-N., Ni F.-D. (2023). Effects of Dysregulated Glucose Metabolism on the Occurrence and ART Outcome of Endometriosis. Eur. J. Med. Res..

[7] Li B., Zhang Y., Zhang L., Zhang L. (2023). Association Between Endometriosis and Metabolic Syndrome: A Cross-Sectional Study Based on the National Health and Nutrition Examination Survey Data. Gynecol. Endocrinol..

[8] Ashish A., Rai S., Mishra S., Maurya A.K., Yadav A.K., Vishwakarma S., Singh R. (2025). Cytokine Profiles and Metabolic Dysregulation in Endometriosis: Insights into Diagnostic and Therapeutic Targets. Mol. Biol. Rep..

[9] Piriyev E., Mennicken C., Schiermeier S., Römer T. (2025). Does BMI Have an Impact on Endometriosis Symptoms and Endometriosis Types According to the #ENZIAN Classification?. J. Clin. Med..

[10] Krasnyi A.M., Sadekova A.A., Smolnova T.Y., Chursin V.V., Buralkina N.A., Chuprynin V.D., Yarotskaya E., Pavlovich S.V., Sukhikh G.T. (2022). The Levels of Ghrelin, Glucagon, Visfatin and Glp-1 Are Decreased in the Peritoneal Fluid of Women with Endometriosis along with the Increased Expression of the CD10 Protease by the Macrophages. Int. J. Mol. Sci..

[11] Eisa N., Barood O. (2026). Tirzepatide Beyond Diabetes and Obesity: Systematic Review and Meta-Analysis of Multisystem Therapeutic Benefits. Endocr. Pract..

[12] Ghaleb J., Khouzami K.K., Nassif N., Attieh P., Al Ajlani M.F., Bou Sleiman J., Khalouf A., Harb F., Azar S., Kannan A. (2025). Unveiling Tirzepatide’s Therapeutic Spectrum: A Dual GIP/GLP-1 Agonist Targeting Metabolic, Neurological, and Cardiovascular Health. Int. J. Endocrinol..

[13] Dong S., Xu Y., Gan R., Zhang A., He J., Tang Q. (2026). Tirzepatide in Metabolic Diseases: Clinical Efficacy and Safety Beyond Diabetes and Obesity. Med. Res. Rev..

[14] Gholiof M., Kalani N., Leonardi M. (2026). Effects of Glucagon-Like Peptide-1 Receptor Agonists (GLP-1RAs) on Endometriosis Symptoms Management and Quality of Life: An International Survey Study. Authorea.

[15] Viana D.P.d.C., Invitti A.L., Schor E. (2025). Tirzepatide as a Potential Disease-Modifying Therapy in Lipedema: A Narrative Review on Bridging Metabolism, Inflammation, and Fibrosis. Int. J. Mol. Sci..

[16] Sarsenova M., Lawarde A., Pathare A.D.S., Saare M., Modhukur V., Soplepmann P., Terasmaa A., Käämbre T., Salumets A., Peters M. (2024). Endometriotic Lesions Exhibit Distinct Metabolic Signature Compared to Paired Eutopic Endometrium at the Single-Cell Level. Commun. Biol..

[17] Guo C., Na X., Guo Z., Jiao J., Yang M., Liang J., Dai W., Na Z., Jiang Z., Li Y. (2026). Metabolic Reprogramming in Endometriosis: Mechanisms and Therapeutic Prospects. J. Adv. Res..

[18] Kobayashi H., Shigetomi H., Imanaka S. (2021). Nonhormonal Therapy for Endometriosis Based on Energy Metabolism Regulation. Reprod. Fertil..

[19] Capobianco A., Rovere-Querini P. (2013). Endometriosis, a Disease of the Macrophage. Front. Immunol..

[20] Ramírez-Pavez T.N., Martínez-Esparza M., Ruiz-Alcaraz A.J., Marín-Sánchez P., Machado-Linde F., García-Peñarrubia P. (2021). The Role of Peritoneal Macrophages in Endometriosis. Int. J. Mol. Sci..

[21] Abramiuk M., Grywalska E., Małkowska P., Sierawska O., Hrynkiewicz R., Niedźwiedzka-Rystwej P. (2022). The Role of the Immune System in the Development of Endometriosis. Cells.

[22] Wang X., Wu N., Xue Q. (2025). Macrophages in Endometriosis: Key Roles and Emerging Therapeutic Opportunities—A Narrative Review. Reprod. Biol. Endocrinol..

[23] Hassanzadeh Makoui M., Fekri S., Hassanzadeh Makoui R., Ansari N., Esmaeilzadeh A. (2025). The Role of Mast Cells in the Development and Advancement of Endometriosis. Am. J. Reprod. Immunol..

[24] Mehdi S.F., Pusapati S., Anwar M.S., Lohana D., Kumar P., Nandula S.A., Nawaz F.K., Tracey K., Roth J., Brownstein M.J. (2023). Glucagon-Like Peptide-1: A Multi-Faceted Anti-Inflammatory Agent. Front. Immunol..

[25] Rahmioglu N., Mortlock S., Ghiasi M., Møller P.L., Stefansdottir L., Galarneau G., Turman C., Danning R., Law M.H., Sapkota Y. (2023). The Genetic Basis of Endometriosis and Comorbidity with Other Pain and Inflammatory Conditions. Nat. Genet..

[26] Yang F., Luo X., Li J., Lei Y., Zeng F., Huang X., Lan Y., Liu R. (2022). Application of Glucagon-Like Peptide-1 Receptor Antagonists in Fibrotic Diseases. Biomed. Pharmacother..

[27] Lu J., Ling X., Liu L., Jiang A., Ren C., Lu C., Yu Z. (2023). Emerging Hallmarks of Endometriosis Metabolism: A Promising Target for the Treatment of Endometriosis. Biochim. Biophys. Acta Mol. Cell Res..

[28] Maddern J., Grundy L., Castro J., Brierley S.M. (2020). Pain in Endometriosis. Front. Cell. Neurosci..

[29] Chen Y., Li T. (2025). Unveiling the Mechanisms of Pain in Endometriosis: Comprehensive Analysis of Inflammatory Sensitization and Therapeutic Potential. Int. J. Mol. Sci..

[30] Machairiotis N., Vasilakaki S., Thomakos N. (2021). Inflammatory Mediators and Pain in Endometriosis: A Systematic Review. Biomedicines.

[31] Gong N., Xiao Q., Zhu B., Zhang C.-Y., Wang Y.-C., Fan H., Ma A.-N., Wang Y.-X. (2014). Activation of Spinal Glucagon-Like Peptide-1 Receptors Specifically Suppresses Pain Hypersensitivity. J. Neurosci..

[32] Ahmad S.F., Carter R.N., Collins F., Greaves E., Morton N.M., Saunders P.T.K., Horne A.W. (2016). Defining the Metabolic Phenotype of Peritoneal Mesothelial Cells from Women with Endometriosis. Endocr. Abstr..

[33] Chang L.-Y., Hou X.-X., Li D.-J., Wang X.-Q. (2025). Metabolite Changes in Patients with Endometriosis: New Potential Diagnostic and Therapeutic Targets. Reprod. Dev. Med..

[34] Lu C., Xu J., Li K., Wang J., Dai Y., Chen Y., Chai R., Xu C., Kang Y. (2024). Chronic Stress Blocks the Endometriosis Immune Response by Metabolic Reprogramming. Int. J. Mol. Sci..

[35] Li Y., Lv X., Jiang M., Jin Z. (2022). Sitagliptin Ameliorates Hypoxia-Induced Damages in Endometrial Stromal Cells: An Implication in Endometriosis. Bioengineered.

[36] Kanda R., Hiraike H., Wada-Hiraike O., Ichinose T., Nagasaka K., Sasajima Y., Ryo E., Fujii T., Osuga Y., Ayabe T. (2018). Expression of the Glucagon-Like Peptide-1 Receptor and Its Role in Regulating Autophagy in Endometrial Cancer. BMC Cancer.

[37] Hogg C., Horne A.W., Greaves E. (2020). Endometriosis-Associated Macrophages: Origin, Phenotype, and Function. Front. Endocrinol..

[38] Henlon Y., Panir K., McIntyre I., Hogg C., Dhami P., Cuff A.O., Senior A., Moolchandani-Adwani N., Courtois E.T., Horne A.W. (2024). Single-Cell Analysis Identifies Distinct Macrophage Phenotypes Associated with Prodisease and Proresolving Functions in the Endometriotic Niche. Proc. Natl. Acad. Sci. USA.

[39] Greygoose E., Metharom P., Kula H., Seckin T.K., Seckin T.A., Ayhan A., Yu Y. (2025). The Estrogen–Immune Interface in Endometriosis. Cells.

[40] Guo C., Huang T., Chen A., Chen X., Wang L., Shen F., Gu X. (2016). Glucagon-like Peptide 1 Improves Insulin Resistance In Vitro through Anti-Inflammation of Macrophages. Braz. J. Med. Biol. Res..

[41] Rahman M.S., Park Y., Hosseinirad H., Shin J.-H., Jeong J.-W. (2025). The Interplay between Endometriosis and Obesity. Trends Endocrinol. Metab..

[42] Pinto da Costa Viana D., Caseri Câmara L., Borges Palau R. (2025). Menopause as a Critical Turning Point in Lipedema: The Estrogen Receptor Imbalance, Intracrine Estrogen, and Adipose Tissue Dysfunction Model. Int. J. Mol. Sci..

[43] Matsuzaki S., Darcha C. (2012). Epithelial to Mesenchymal Transition-Like and Mesenchymal to Epithelial Transition-Like Processes Might Be Involved in the Pathogenesis of Pelvic Endometriosis. Hum. Reprod..

[44] Chen M., Zhou Y., Xu H., Hill C., Ewing R.M., He D., Zhang X., Wang Y. (2020). Bioinformatic Analysis Reveals the Importance of Epithelial-Mesenchymal Transition in the Development of Endometriosis. Sci. Rep..

[45] Zhang Q., Duan J., Olson M., Fazleabas A., Guo S.-W. (2016). Cellular Changes Consistent with Epithelial-Mesenchymal Transition and Fibroblast-to-Myofibroblast Transdifferentiation in the Progression of Experimental Endometriosis in Baboons. Reprod. Sci..

[46] Ma L., Andrieu T., McKinnon B., Duempelmann L., Peng R.-W., Wotzkow C., Müller C., Mueller M.D. (2021). Epithelial-to-Mesenchymal Transition Contributes to the Downregulation of Progesterone Receptor Expression in Endometriosis Lesions. J. Steroid Biochem. Mol. Biol..

[47] Appleyard C.B., Flores I., Torres-Reverón A. (2020). The Link Between Stress and Endometriosis: From Animal Models to the Clinical Scenario. Reprod. Sci..

[48] Ma X.L., Ding Y., Wu L.M., Wang J., Zhang J.J., Liu H.M. (2021). The Glucagon-Like Peptide-1 (GLP-1) Analog Exenatide Ameliorates Intrauterine Adhesions in Mice. Peptides.

[49] Morotti M., Vincent K., Brawn J., Zondervan K.T., Becker C.M. (2014). Peripheral Changes in Endometriosis-Associated Pain. Hum. Reprod. Update.

[50] Morotti M., Vincent K., Becker C.M. (2017). Mechanisms of Pain in Endometriosis. Eur. J. Obstet. Gynecol. Reprod. Biol..

[51] Coxon L., Demetriou L., Vincent K. (2024). Current Developments in Endometriosis-Associated Pain. Cell Rep. Med..

[52] Coxon L., Evans E., Vincent K. (2023). Endometriosis—A Painful Disease. Curr. Opin. Anaesthesiol..

[53] Olteanu I.-L., Pușcașu C., Andrei C., Zanfirescu A. (2026). Hormonal Dysregulation and Neuroinflammation in Endometriosis: Convergent Druggable Pathways. Curr. Issues Mol. Biol..

[54] McMahon S.B., La Russa F., Bennett D.L. (2015). Crosstalk between the Nociceptive and Immune Systems in Host Defence and Disease. Nat. Rev. Neurosci..

[55] Dutta M., Singh B., Joshi M., Das D., Subramani E., Maan M., Jana S.K., Sharma U., Das S., Dasgupta S. (2018). Metabolomics Reveals Perturbations in Endometrium and Serum of Minimal and Mild Endometriosis. Sci. Rep..

[56] He Y., Xu B., Zhang M., Chen D., Wu S., Gao J., Liu Y., Zhang Z., Kuang J., Fang Q. (2025). Advances in GLP-1 Receptor Agonists for Pain Treatment and Their Future Potential. J. Headache Pain.

[57] Jing F., Zeng Y., Yu Q.L., Fu C.J. (2025). The Role of GLP-1 Receptor in Pain Disorders and Its Pharmacological Properties. Eur. J. Pharmacol..

[58] Halloum W., Al Dughem Y., Beier D., Pellesi L. (2024). Glucagon-like Peptide-1 (GLP-1) Receptor Agonists for Headache and Pain Disorders: A Systematic Review. J. Headache Pain.

[59] Mitchell J.L., Lyons H.S., Walker J.K., Yiangou A., Grech O., Alimajstorovic Z., Greig N.H., Li Y., Tsermoulas G., Brock K. (2023). The Effect of GLP-1RA Exenatide on Idiopathic Intracranial Hypertension: A Randomized Clinical Trial. Brain.

[60] Bliddal H., Bays H., Czernichow S., Uddén Hemmingsson J., Hjelmesæth J., Hoffmann Morville T., Koroleva A., Skov Neergaard J., Sánchez Pérez M.V., Wharton S. (2024). Once-Weekly Semaglutide in Persons with Obesity and Knee Osteoarthritis. N. Engl. J. Med..

[61] Krajnc N., Itariu B., Macher S., Marik W., Harreiter J., Michl M., Novak K., Wöber C., Pemp B., Bsteh G. (2023). Treatment with GLP-1 Receptor Agonists Is Associated with Significant Weight Loss and Favorable Headache Outcomes in Idiopathic Intracranial Hypertension. J. Headache Pain.

[62] Zhu H., Zhou L., Wang Q., Cai Q., Yang F., Jin H., Chen Y., Liu Y., Wang Y., Wang S. (2023). Glucagon-Like Peptide-1 Receptor Agonists as a Disease-Modifying Therapy for Knee Osteoarthritis Mediated by Weight Loss: Findings from the Shanghai Osteoarthritis Cohort. Ann. Rheum. Dis..

[63] Simancas-Racines D., Jiménez-Flores E., Montalvan M., Horowitz R., Araujo V., Reytor-González C. (2026). Endometriosis as a Systemic and Complex Disease: Toward Phenotype-Based Classification and Personalized Therapy. Int. J. Mol. Sci..

[64] Imperiale L., Nisolle M., Noël J.-C., Fastrez M. (2023). Three Types of Endometriosis: Pathogenesis, Diagnosis and Treatment. State of the Art. J. Clin. Med..

[65] Shin S., Chung Y.-J., Moon S.W., Choi E.J., Kim M.-R., Chung Y.-J., Lee S.H. (2023). Single-Cell Profiling Identifies Distinct Hormonal, Immunologic, and Inflammatory Signatures of Endometriosis-Constituting Cells. J. Pathol..

[66] Vissers G., Giacomozzi M., Verdurmen W., Peek R., Nap A. (2024). The Role of Fibrosis in Endometriosis: A Systematic Review. Hum. Reprod. Update.

[67] Viana D.P.d.C., Invitti A.L., Schor E. (2026). Lipedema and Dynapenia: Inflammatory Myosteatosis as a Mechanistic Link Between Tissue Expansion and Muscle Dysfunction. Int. J. Mol. Sci..

[68] Jalleh R.J., Plummer M.P., Marathe C.S., Umapathysivam M.M., Quast D.R., Rayner C.K., Jones K.L., Wu T., Horowitz M., Nauck M.A. (2024). Clinical Consequences of Delayed Gastric Emptying with GLP-1 Receptor Agonists and Tirzepatide. J. Clin. Endocrinol. Metab..

[69] Khan D., Ojo O.O., Woodward O.R., Lewis J.E., Sridhar A., Gribble F.M., Reimann F., Flatt P.R., Moffett R.C. (2022). Evidence for Involvement of GIP and GLP-1 Receptors and the Gut-Gonadal Axis in Regulating Female Reproductive Function in Mice. Biomolecules.

[70] Couldwell M., Tidwell A.J., Taylor A.E. (2025). Effect of GLP1 Agonists on Reproduction. J. Clin. Endocrinol. Metab..

[71] Coskun T., Sloop K.W., Loghin C., Alsina-Fernandez J., Urva S., Bokvist K.B., Cui X., Briere D.A., Cabrera O., Roell W.C. (2018). LY3298176, a Novel Dual GIP and GLP-1 Receptor Agonist for the Treatment of Type 2 Diabetes Mellitus: From Discovery to Clinical Proof of Concept. Mol. Metab..

